# Supplementary material for: Self-Assembly Strategy for Synthesis of WO3@TCN Heterojunction: Efficient for Photocatalytic Degradation and Hydrogen Production via Water Splitting
Source: Molecules. 2025 Jan 17;30(2):379. doi: 10.3390/molecules30020379 (PMC11767450; doi:10.3390/molecules30020379)
Supplement: Supplementary file 1 [file molecules-30-00379-s001.zip › molecules-3419957-supplementary.pdf]

# Supporting Information

## Self-assembly strategy for synthesis of $WO_3$@TCN heterojunction: Efficient for photocatalytic degradation and hydrogen production via water splitting

Li Zhou, Wenjie Zhang, Zezhao Huang, Feng Hu, Peng Li *and Xiaoquan Yao *

Department of Applied Chemistry, School of Material Science and Technology, Nanjing University of Aeronautics and Astronautics, Nanjing 210016, P. R. China.

*Corresponding author：
E-mail addresses: yaoxq@nuaa.edu.cn (X. Yao), lpeng@nuaa.edu.cn (P. Li),

# Table of Contents


| | | |
|---|---|---|
| **Experimental section** | Measurement and Characterization | S3 |
| | DFT Theoretical Calculations | S4 |
| **Table S1**. | Elemental content and ratios of samples | S5 |
| **Table S2** | Specific surface area, pore size and pore volume data sheets | S5 |
| **Table S3** | Comparison of photocatalytic degradation efficiency of tetracycline with 3%$WO_3$@TCN and other g-$C_3N_4$ samples | S5 |
| **Figure S1.** | EDS elemental mapping images of 3%$WO_3$@TCN | S6 |
| **Figure S2.** | Photoluminescence spectra of TCN and 3%$WO_3$@TCN | S7 |
| **Figure S3.** | 3%$WO_3$@TCN photocatalytic degradation of tetracycline in parallel experiments | S8 |
| **Figure S4.** | Stability experiments of photocatalytic degradation of tetracycline of 3%$WO_3$@TCN | S9 |
| **Figure S5.** | EPR spectra of DMPO-$^{\bullet}O_2^{-}$ | S10 |
| **Figure S6.** | Photocatalytic hydrogen evolution from water splitting on $WO_3$、TCN and 3%$WO_3$@TCN | S11 |
| **Reference** | | S12 |

## Experimental section:

### Measurement and Characterization

The crystal phase, photoelectric properties, elemental compositions, and morphologies were performed using an X-ray diffraction (XRD) spectroscope (Bruker D8 ADVANCE), UV–vis spectroscopy (JP Shimadzu Co., Ltd), scanning electron microscope (SEM) (Mira 4, Tescan) with energy-dispersed spectroscopy (EDS, Oxford Ultim Max65), and X-ray photoelectron spectroscopy (XPS) (250Xi, Thermo Fisher). Fourier transform infrared spectra (FT-IR) were analyzed by Thermo Scientific Nicolet iS20. The specific surface areas were determined with a surface area analyzer (ASAP 2020 Micropore System, Micromeritics Instrument Corporation, USA) by the Brunauer-Emmett-Teller (BET) method. The photo-generated charge carrier separation was recorded by Steady-state Photoluminescence (PL) spectra (Cray Eclipse, Varian, US).

## DFT Theoretical Calculations

Based on the DFT theory, the figure of merit was calculated in this paper using the Vienna analog package (VASP) for first-principle DFT of composite photocatalysts. The truncation energy of the plane wave was set to 450 eV. In order to balance the geometry and optimize the lattice structure, the energy and force converged to $10^{-4}$ eV and 0.03 eV, respectively, with a K-point of 221. The crystal plane of $WO_3$ (010) and g-$C_3N_4$ (001) were selected for calculating. The P-space group was selected for $WO_3$ (a = 7.384 Å, b = 7.512 Å, and c = 3.846 Å), which corresponds to α = 90.00°, β = 90.00° , and γ = 90.00° to model the correlation. In addition, the crystal parameters of $WO_3$ and g-$C_3N_4$ materials were obtained from MDI Jade 6.5.

**Table S1**. Elemental Content and Ratios of Samples Obtained from Elemental Analysis and XPS Data

| Catalysist | elemental content [a] (wt %) | | | |
|---|---|---|---|---|
| | **C** | **N** | **O** | **W** |
| TCN | 45.39 | 54.61 | | |
| 3%$WO_3$@TCN | 43.02 | 54.06 | 2.84 | 0.08 |

[a] Elemental analysis results.

**Table S2**. Specific surface area, pore size and pore volume data sheets for samples

| **Catalysist** | **$S_{BET}$( $m^2/g$)** | **Pore diameter(nm)** | **Pore volume ($cm^3/g$)** |
|---|---|---|---|
| $WO_3$ | 10.16 | 7.80 | 0.030 |
| TCN | 19.39 | 6.24 | 0.020 |
| 3% $WO_3$@TCN | 26.51 | 8.15 | 0.053 |

**Table S3.** Comparison of photocatalytic degradation efficiency of tetracycline with 3%$WO_3$@TCN and other g-$C_3N_4$ samples

| **Catalyst** | **Dosage （mg）** | **Light source** | **Initial amount** | **Time （min）** | **Removal （%）** |
|---|---|---|---|---|---|
| Mpg-$C_3N_4$-ZIF-8[1] | 50 | 500W,Xe （λ≥ 420nm） | 40 mg/L | 180 | 74.8 |
| Ag/g-$C_3N_4$[2] | 50 | 300W, Xenon （λ≥ 420 nm） | 20 mg/L | 120 | 83 |
| $BiVO_4$/g-$C_3N_4$[3] | 20 | 300W, Xenon | 20 mg/L | 60 | 56 |
| CeNCN[4] | 10 | 300W, Xenon （λ≥420 nm） | 10 mg/L | 60 | 80.09 |
| Tubular g-$C_3N_4$[5] | 20 | 300W, Xenon （λ≥420 nm） | 10 mg/L | 60 | 50 |

| | | | | | |
|---|---|---|---|---|---|
| 3%$WO_3$@TCN | 10 | 300W, Xenon（λ≥ 420 nm） | 20 mg/L | 80 | 86.5 |

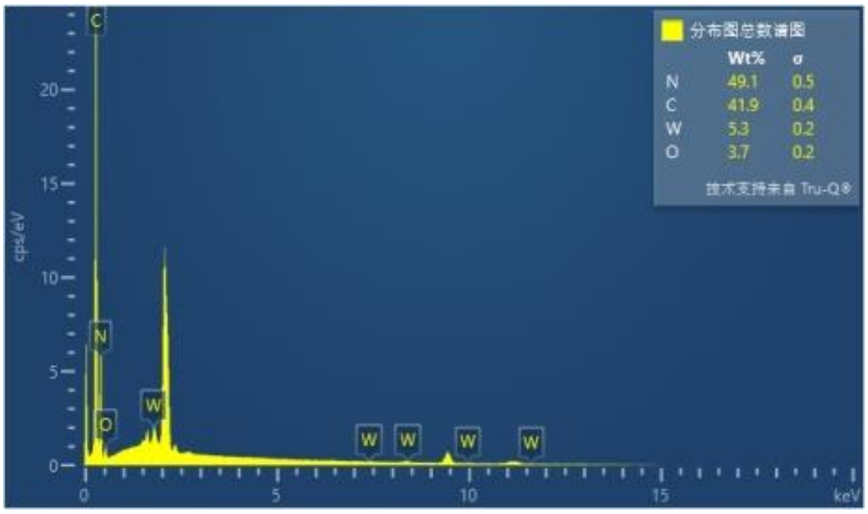


**Figure S1.** EDS elemental mapping images of 3%$WO_3$@TCN.

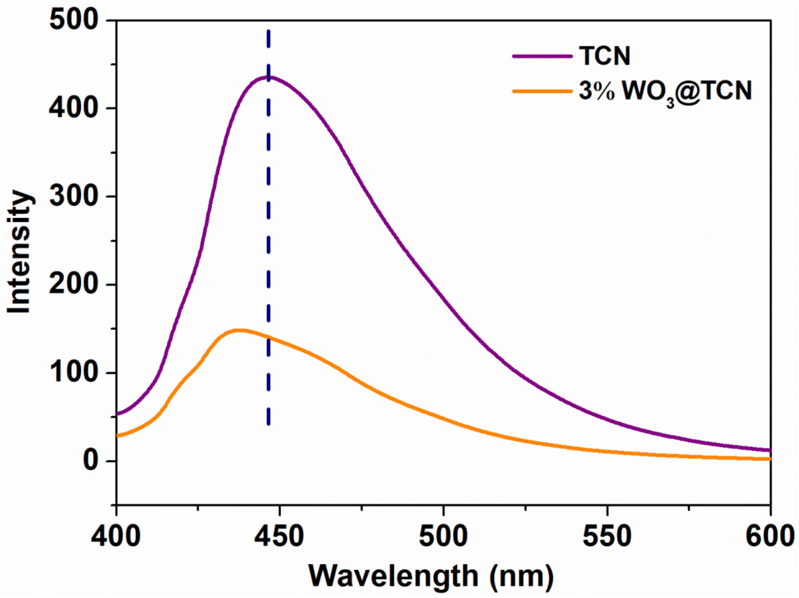


**Figure S2.** Photoluminescence spectra of TCN and 3%$WO_3$@TCN.

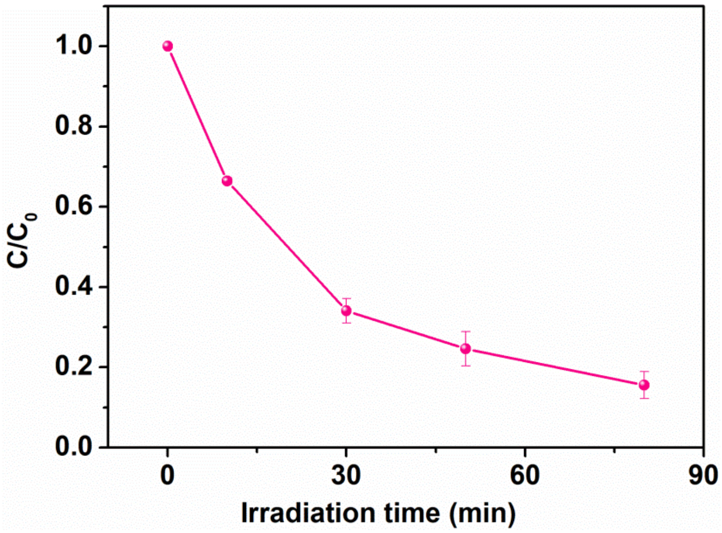


**Figure S3.** 3%$WO_3$@TCN photocatalytic degradation of tetracycline in parallel experiments

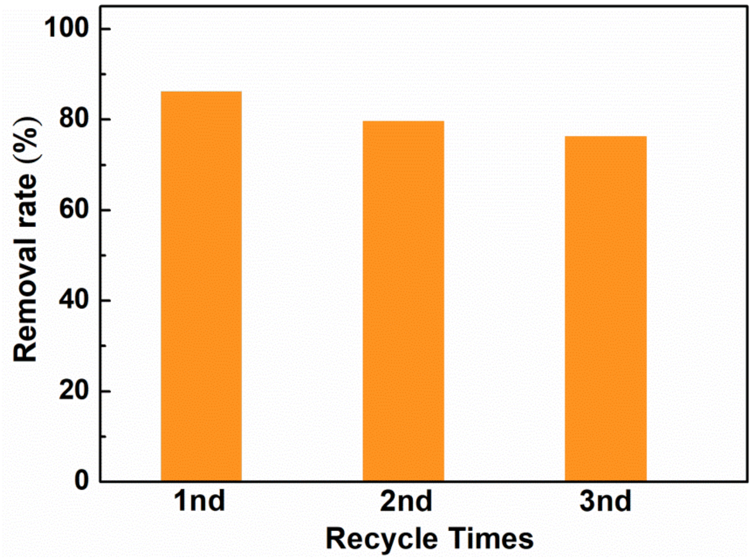


**Figure S4.** Stability experiments of photocatalytic degradation of tetracycline of 3%$WO_3$@TCN.

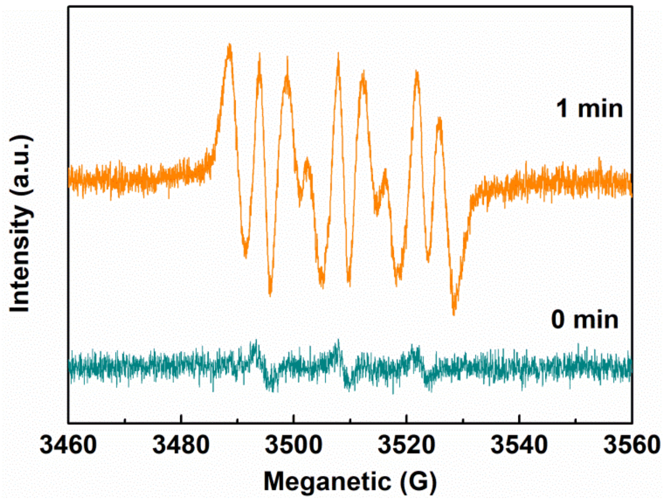


**Figure S5.** EPR spectra of DMPO-$^{\bullet}O_2^-$ in methanol dispersion in the presence of 3%$WO_3$@TCN

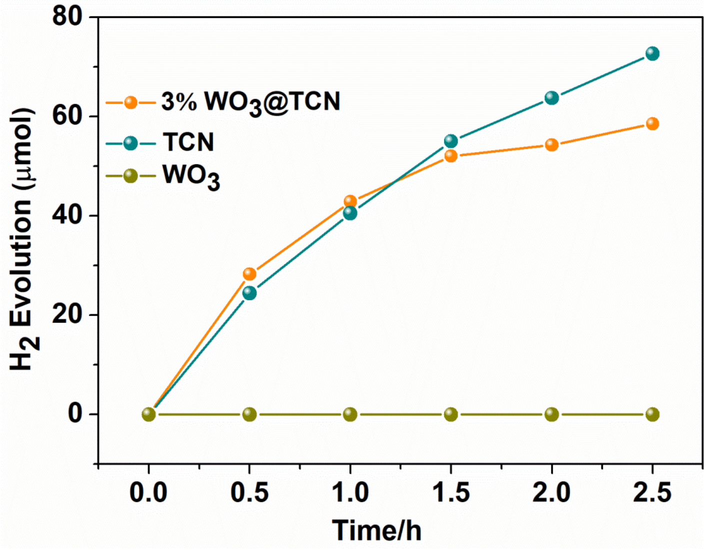


**Figure S6.** Photocatalytic hydrogen evolution from water splitting on $WO_3$、TCN and 3%$WO_3$@TCN with Pt as the cocatalyst and triethanolamine as the electron donor, under Xe lamp irradiation.

## Reference

[1] Li D, Liu H, Niu C, et al. Mpg-$C_3N_4$-ZIF-8 composites for the degradation of tetracycline hydrochloride using visible light. Water Science Technology, 2019, 80(11): 2206-2217.

[2] Xu W, Lai S, Pillai S C, et al. Visible light photocatalytic degradation of tetracycline with porous Ag/graphite carbon nitride plasmonic composite: Degradation pathways and mechanism. Journal of Colloid and Interface Science, 2020, 574: 110-121.

[3] Zhang X, Xie X, Li J, et al. Type II heterojunction formed between 010 or 012 facets dominated bismuth vanadium oxide and carbon nitride to enhance the photocatalytic degradation of tetracycline. International Journal of Environmental Research and Public Health, 2022, 19(22): 14770.

[4] Xu F, An N, Lai C, et al. Nitrogen-doping coupled with cerium oxide loading co-modified graphitic carbon nitride for highly enhanced photocatalytic degradation of tetracycline under visible light. Chemosphere, 2022, 293: 133648.

[5] M Cao, J Zuo, Y Huang, Z Liu. Synthesis of tubular g-$C_3N_4$ via a $H_2SO_4$-assisted precursor self-assembly strategy for enhanced photocatalytic degradation of organic pollutant. Journal of Materials Science: Materials in Electronics, 2020, 31: 2022-2029.
